# Antenatal and postnatal corticosteroid and resuscitation induced lung injury in preterm sheep

**DOI:** 10.1186/1465-9921-10-124

**Published:** 2009-12-15

**Authors:** Noah H Hillman, J Jane Pillow, Molly K Ball, Graeme R Polglase, Suhas G Kallapur, Alan H Jobe

**Affiliations:** 1 Cincinnati Children's Hospital Medical Center, Division of Pulmonary Biology, Cincinnati, OH 45236, USA; 2 School of Women's and Infants' Health, The University of Western Australia, Perth, WA 6009, Australia; 3 Northwestern University, Department of Neonatology, Chicago, IL 60614, USA

## Abstract

**Background:**

Initiation of ventilation using high tidal volumes in preterm lambs causes lung injury and inflammation. Antenatal corticosteroids mature the lungs of preterm infants and postnatal corticosteroids are used to treat bronchopulmonary dysplasia.

**Objective:**

To test if antenatal or postnatal corticosteroids would decrease resuscitation induced lung injury.

**Methods:**

129 d gestational age lambs (n = 5-8/gp; term = 150 d) were operatively delivered and ventilated after exposure to either 1) no medication, 2) antenatal maternal IM Betamethasone 0.5 mg/kg 24 h prior to delivery, 3) 0.5 mg/kg Dexamethasone IV at delivery or 4) Cortisol 2 mg/kg IV at delivery. Lambs then were ventilated with no PEEP and escalating tidal volumes (*V*_T_) to 15 mL/kg for 15 min and then given surfactant. The lambs were ventilated with *V*_T _8 mL/kg and PEEP 5 cmH_2_0 for 2 h 45 min.

**Results:**

High V_T _ventilation caused a deterioration of lung physiology, lung inflammation and injury. Antenatal betamethasone improved ventilation, decreased inflammatory cytokine mRNA expression and alveolar protein leak, but did not prevent neutrophil influx. Postnatal dexamethasone decreased pro-inflammatory cytokine expression, but had no beneficial effect on ventilation, and postnatal cortisol had no effect. Ventilation increased liver serum amyloid mRNA expression, which was unaffected by corticosteroids.

**Conclusions:**

Antenatal betamethasone decreased lung injury without decreasing lung inflammatory cells or systemic acute phase responses. Postnatal dexamethasone or cortisol, at the doses tested, did not have important effects on lung function or injury, suggesting that corticosteroids given at birth will not decrease resuscitation mediated injury.

## Introduction

The majority of very low birth weight infants are intubated and receive mechanical ventilation at birth [1]. A few large tidal volume breaths can inactivate surfactant [2], and initiation of ventilation with large tidal volumes activates an inflammatory cascade in the medium and small airways [3]. In surfactant deficient animals, normal tidal volume ventilation from birth initiates an inflammatory cascade characterized by inflammatory cell influx into the lungs, increased alveolar protein, inflammatory cytokine mRNA induction, and systemic acute phase inflammatory responses [4]. Mechanical ventilation is associated with an increased risk of bronchopulmonary dysplasia (BPD), and alternatives to delivery room intubation and ventilation tend to decrease BPD [5,6]. Lung inflammation is a major contributor to the pathophysiology of BPD [7].

Antenatal corticosteroids have pleotrophic effects that include induced lung maturation and decreased neonatal mortality, respiratory distress syndrome (RDS), intraventricular hemorrhage, and necrotizing enterocolitis, but no decrease in BPD [8]. Antenatal corticosteroids also increase the antioxidant defenses of very low birth weight infants and preterm sheep [9,10]. Antenatal corticosteroids are currently recommended for women 24 to 34 weeks gestation at risk for preterm delivery [11]. Postnatal corticosteroids, primarily dexamethasone, are used to wean infants from ventilatory support and to decrease BPD [12]. Although some infants exposed to postnatal corticosteroids have impaired neurodevelopment, infants with high risk for BPD benefit from weaning from the ventilator and a decrease in BPD [13]. Hydrocortisone, used to treat relative adrenal insufficiency in premature infants, decreased the incidence of BPD in infants exposed to chorioamnionitis [14]. The presumed beneficial effects of corticosteroids in BPD are to decrease lung inflammation and microvascular permeability [15].

The initiation of ventilation at birth with large tidal volumes for 15 minutes causes an acute stretch induced lung injury and a systemic inflammatory response [16]. Ventilation of preterm lambs activates Early growth protein 1 (Egr-1) and other pro-inflammatory signaling pathways [17] that are inhibited by corticosteroids [18]. Corticosteroids decrease stretch induced lung injury in adult animals [19]. Corticosteroids given prior to cardiopulmonary bypass also decrease systemic inflammation and acute phase responses [20]. Since different corticosteroids have different potencies and glucocorticoid effects [21], we have tested the common corticosteroids used clinically in preterm infants. We hypothesized that antenatal betamethasone or postnatal dexamethasone or cortisol will decrease lung and systemic injury caused from initiating ventilation with high V_T _in preterm sheep.

## Materials and methods

The animal studies were performed in Perth, Western Australia after approval from the animal care and use committees at Cincinnati Children's Hospital and the University of Western Australia.

### Treatment Groups

Time-mated 129 d gestational age preterm lambs (term ~150 d) were operatively delivered, a tracheostomy performed, and lung fluid removed [4]. An external jugular catheter was placed prior to clamping the umbilical cord. Lambs were randomly assigned to 5 experimental groups: 1) No steroids (Injury), 2) Maternal betamethasone 0.5 mg/kg IM to the ewe 24 h before delivery (Injury + Beta), 3) dexamethasone 0.5 mg/kg IV following cord clamping and prior to ventilation (Injury + Dex), 4) cortisol 2 mg/kg IV following cord clamping and prior to ventilation (Injury + Cortisol), or 5) non-ventilated fetal controls. The maternal betamethasone dose was the effective dose for lung maturation in sheep [22] and is similar to the dose given to pregnant women at risk for preterm birth. The 0.5 mg/kg Dex dose is the high dose used clinically for treating infants with BPD [12]. The cortisol dose (2 mg/kg) is higher than used in clinical trials [14], but equivalent to dose given over 24 h for hypotension in preterm infants [23]. The higher doses of postnatal corticosteroids were chosen to evaluate their anti-inflammatory effects.

#### Lung Injury for 15 Minutes

Ventilation was initiated (rate 40 breaths/min, inspiratory time 0.7 s, FiO_2 _0.40) with a Drager BL8000+ ventilator (Drager, Lubeck, Germany) using a time-cycled, volume-guarantee mode and 8 L/min flow with heated and humidified gas and no positive end expiratory pressure (PEEP). Tidal volumes (*V*_T_) were escalated to achieve the target *V*_T _of 8-10 mL/kg at 5 minutes, 12-15 mL/kg by 10 minutes and 15 mL/kg at 15 minutes to injure the lungs [16]. Lambs were treated with 100 mg/kg porcine surfactant at 15 min of age (Curosurf^®^, kindly provided by Chiesi Pharmaceuticals, Italy). The umbilical artery was catheterized for blood gas sampling. An umbilical vein catheters were placed for continuous infusion of Remifentanil (0.05 μg/kg/h; Ultiva, Glaxo Smith Kline, Victoria, Australia) and Propofol (0.1 mg/kg/h; Repose, Norbrook Laboratories, Victoria, Australia).

#### Subsequent Ventilation

Following surfactant treatment at 15 min of age, the volume guarantee ventilation mode was decreased to 7 mL/kg and lambs were ventilated for the remaining study period (2 h 45 min) with a heated and humidified oxygen and air mixture (40 breaths/min, PEEP 5 cmH_2_0, inspiratory time 0.7 s, Fi0_2 _0.40). A *P*aCO_2 _of 50 mmHg was targeted by adjusting the volume guarantee. FiO_2 _was adjusted to maintain a oxygen saturation on pulse oximetry of greater than 90%. Blood-gas status and ventilation variables were recorded every 15 minutes for first hour, then every 30 minutes. Ventilation Efficiency index (VEI) was calculated as 3800/((PIP-PEEP) ventilator rate • *P*aCO_2_). Oxygenation Index (OI) was calculated as FiO_2 _• Mean Airway Pressure • 100/*P*aO_2_. Lambs were killed with a lethal intravenous dose of pentobarbital (100 mg/kg, Valabarb, Jurox, Rutherford, NSW, Australia) 3 h after delivery.

### Lung Processing and BAL Analysis

The lungs were weighed, and bronchoalveolar lavage (BAL) was recovered by saline lavage of the left lung[24]. Tissues from the left lung were snap frozen for RNA analysis. The right upper lobe was inflation fixed with 10% formalin [24]. Injury was scored on blinded H&E stained tissue. Ten random high power fields were scored on a 0 to 2 scale for thickness of mesenchyme, hemorrhage, inflammation, and epithelial sloughing (total 8 points)[3]. Cytospins of BAL were used for differential cell counts of neutrophils, monocytes, or epithelial cells [25].

#### Immunohistochemistry

Immunostaining protocols were used as reported[25,26]. Paraffin sections (5 μm) of formalin fixed tissue were pre-treated with 3% hydrogen peroxide to inactivate endogenous peroxidases. The sections were incubated with anti-human Egr-1 1:250 dilution (Santa Cruz, USA) in 4% normal goat serum overnight, followed by biotin labeled secondary antibody. Immunostaining was visualized by Vectastain ABC peroxidase Elite kit to detect the antigen:antibody complexes (Vector Laboratories Inc). The antigen detection was enhanced with nickel-DAB, followed by TRIS-cobalt and the nuclei were counterstained with eosin.

#### RNase protection assays

Total RNA was isolated using a modified Chomzynski method [27], and 10 μg of total lung RNA was used for RNase protection assays using sheep-specific riboprobes for IL-1β, IL-6, MCP-1, HSP70, Egr-1, and L32 [28-30]. Solution hybridization was performed in 80% deionized formamide, 0.4 M NaCl, 2 mM EDTA, and 0.04 M PIPES, pH 6.6, using a molar excess of [^32^P]UTP-labeled probes for 16 h at 56°C. Single-stranded RNA was digested with RNase A/T1 (Pharmingen, San Diego, CA). RNase was inactivated, and the protected RNA was precipitated using RNAse inactivation buffer (Ambion, Austin, TX). L32 (ribosomal protein mRNA) was used as an internal control for loading[30]. The protected fragments were resolved on 6% polyacrylamide 8 mol/L urea gels, visualized by autoradiography, and quantified on a Phospho Imager using ImageQuant version 1.2 software (Molecular Dynamics, Sunnyvale, CA).

#### *In situ *hybridization

Digoxigenin-labeled riboprobes for *In situ *localization (sense and anti-sense) were synthesized from cDNA templates using DIG RNA labeling kits (Roche, USA) and diluted in hybridization buffer to a final concentration of 1 ug/mL. The sections were pre-treated with 4% paraformaldehyde, proteinase K treated, and hybridized with the probe overnight at 49°C. Sections were washed with formamide then treated with RNase A (100 μg/mL), washed and blocked with 10% horse serum. Following incubation overnight at 4°C with anti-Digoxigenin antibody (Roche, USA) and then washing, the slides were developed with NBT-BCIP (Roche, USA) in dark cases. The slides were monitored for color development, then stopped with Tris EDTA buffer. Controls for specificity of ribo-probe binding included use of the homologous (sense) probe.

### Statistics

All values are expressed as means ± SD or individual values plus mean. Comparisons between intervention groups were made with two-tailed Mann-Whitney nonparametric tests, Welch t-tests, or ANOVA where appropriate. Significance was accepted at p < 0.05.

## Results

**Figure 1:**
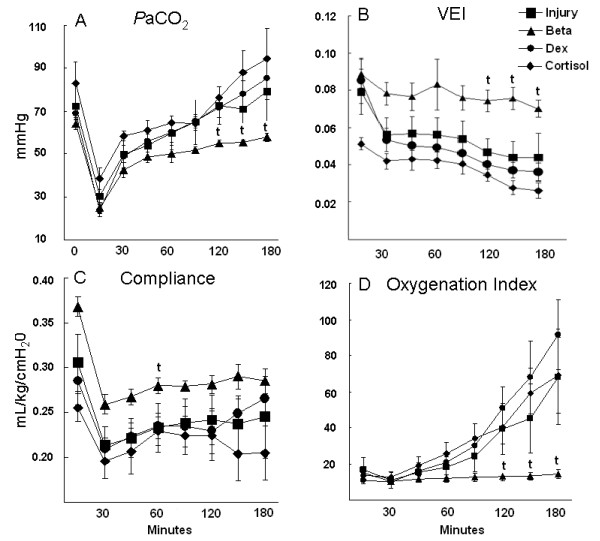
**Pulmonary outcomes**. There were no differences between Injury, Dexamethasone (Dex), and Cortisol groups. (A) *P*aCO_2 _decreased similarly in all groups at 15 min after the initial high V_T _stretch injury, then increased with continued ventilation. *P*aCO_2 _was lower after 120 min in the betamethasone group (Beta) relative to the injury group. (B) Ventilation efficiency index (VEI) decreased with time of ventilation, indicating progressive injury, with less decrease in the Beta group. (C) Dynamic compliance decreased following the stretch injury, with less decrease at 60 min for the Beta group. (D) Oxygenation index increased (indicating deterioration in oxygenation) with ventilation in all groups except the Beta group. t p < 0.05 vs Injury group.

The lambs had similar birth weights, tidal volumes, and peak inspiratory pressures at 15 minutes (Table 1). Although the Injury + Beta animals were the only group to achieve the target V_T _of 15 mL/kg, the lambs exposed to antenatal betamethasone tolerated injurious ventilation better than lambs receiving no steroids. The betamethasone exposed lambs had more stable PaCO_2_, oxygenation index and ventilation efficiency index values throughout the 2 h 45 min ventilation period than did the other groups (Figure 1). Neither postnatal dexamethasone nor cortisol prevented increases in PaCO_2_, decreased oxygenation and overall deterioration of ventilation, despite relatively stable compliance values (Figure 1).

**Table 1:** Description of animals

		Values during V_T _injury					Values at 3 h	
**Groups**	**N**	**BW** **(Kg)**	**V_T _5 min** **(mL/kg)**	**V_T _10 min** **(mL/kg)**	**V_T _15 min** **(mL/kg)**	**PIP 15 min** **(cmH_2 _O)**	**V_T_** **(mL/kg)**	**PIP** **(cmH_2 _O)**

**Unventilated controls**	8	3.1 ± 0.2	-	-	-	-	-	-

**Injury**	8	3.0 ± 0.3	6.9 ± 1.0	9.7 ± 1.5	13.2 ± 1.8	45 ± 8	7.8 ± 1.9	33 ± 9

**Injury + Betamethasone**	8	3.2 ± 0.4	8.0 ± 0.2	10.8 ± 0.3	15.1 ± 0.2^t^	41 ± 4	6.9 ± 1.0	24 ± 4^t^

**Injury + Dexamethasone**	8	2.9 ± 0.4	7.5 ± 1.0	10.5 ± 0.7	13.3 ± 1.9	48 ± 5	7.4 ± 3.1	32 ± 4

**Injury + Cortisol**	5	3.2 ± 0.4	7.3 ± 1.3	9.6 ± 1.7	11.8 ± 2.6	46 ± 8	7.8 ± 1.7	39 ± 4

Mean ± SD. t p < 0.05 vs Injury. BW is birth weight, BAL is bronchoalveolar lavage.

All lambs had increased BAL protein compared to unventilated controls (Table 2). Antenatal betamethasone decreased protein in BAL, but had no effect on the number of inflammatory cells recovered by BAL. Postnatal dexamethasone or cortisol did not change BAL protein or inflammatory cells relative to ventilated controls. Injury scores of betamethasone and dexamethasone exposed lungs showed decreased injury compared to Injury group (Table 2). The betamethasone group, compared to Injury animals, had decreased inflammatory cells and airway thickness of mesenchyme on Injury scoring.

**Table 2:** Markers of Lung and Systemic Injury and Inflammation

Group	N	BAL Protein (mg/kg)	Injury Score (0 ut of 8)	BAL Neutrophils/kg ×10^6^	Liver SAA3 mRNA^1^
**Unventilated Controls**	8	25 ± 12	1.5 ± 0.6	0.1 ± 0.3	1.0 ± 0.05

**Injury**	8	107 ± 24*	5.0 ± 0.9*	23.0 ± 14.9*	3.8 ± 1.4*

**Injury + Beta**	8	60 ± 16* ^t^	3.4 ± 1.0* ^t^	14.5 ± 17.7*	5.4 ± 1.9*

**Injury + Dex**	8	119 ± 32*	3.5 ± 1.0* ^t^	22.0 ± 10.9*	7.2 ± 1.8*

**Injury + Cortisol**	5	97 ± 21*	4.7 ± 0.8*	17.3 ± 11.3*	7.2 ± 1.5*

*p < 0.05 vs Controls, ^t ^p < 0.05 vs Injury. BAL is bronchoalveolar lavage. SAA3 is serum amyloid A3. 1. expressed relative to a relative value of 1 for unventilated controls.

### Lung Cytokines and Acute Phase Reactants

**Figure 2:**
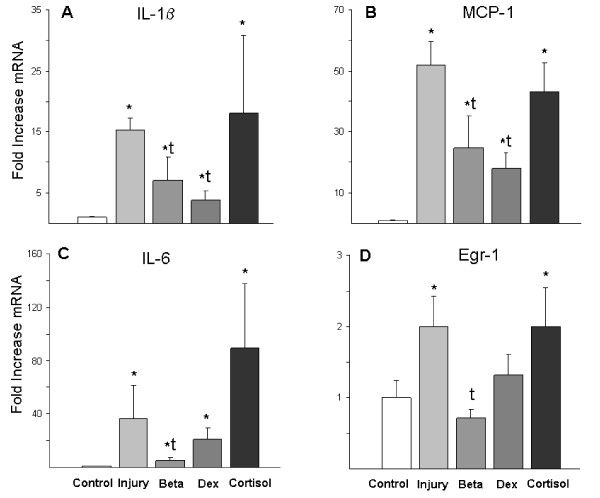
**Cytokines and Early Growth Response Protein 1 mRNA in lung tissue**. (A) IL-1β mRNA and (B) Monocyte chemotactic protein 1 (MCP-1) mRNA increased with the stretch injury and ventilation in all groups relative to unventilated controls. Il-1β and MCP-1 were decreased by Betamethasone (Beta) and Dexamethasone (Dex) compared to the Injury group. (C) The increase in IL-6 mRNA with ventilation was suppressed by Beta. (D) Egr-1 mRNA increased in the Injury and Cortisol groups. Cytokine mRNA was normalized to L32 mRNA (loading control). All values reported as fold increases compared with control animals, normalized to 1. *p < 0.05 vs Controls. ^t ^p < 0.05 vs Injury group

The initial stretch injury increased IL-1β, IL-6, monocyte chemotactic protein 1 (MCP-1), and early response protein 1 (Egr-1) mRNA in the lungs at 3 h (Figure 2). Consistent with the lower lung injury score, betamethasone treatment reduced cytokine production compared to the injury animals. Postnatal dexamethasone decreased lung IL-1β and MCP-1mRNA, but not IL-6 or Egr-1. Cortisol had no effect on lung cytokine mRNA.

**Figure 3:**
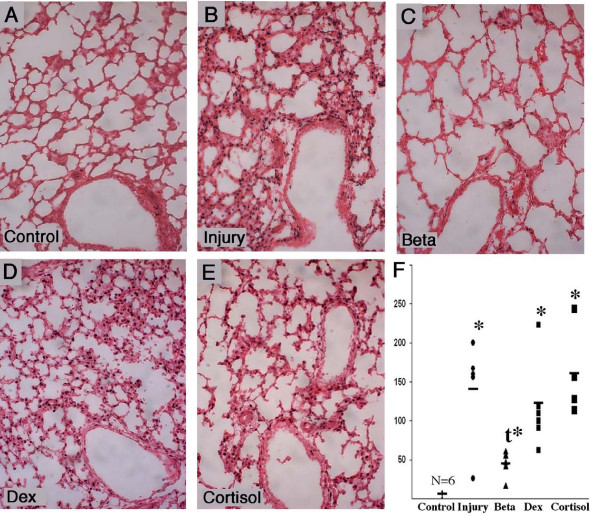
**Early Growth Response Protein 1 increased with stretch injury and ventilation**. (A) Control animals show minimal staining around blood vessels. (B, D, E) The injurious ventilation increased Egr-1 protein surrounding airways, and these increases were not affected by Dex or cortisol. (C) The betamethasone group (Beta) had moderate staining around airways. (F) Semi-quantitative analysis of positive cells per high powered field demonstrated decreased staining in Beta group compared to injurious ventilation. * p < 0.05 vs control, ^t ^p < 0.05 vs Injury group.

Lung Egr-1 mRNA increased about 2 fold in injury group and cortisol groups, but did not change with betamethasone or dexamethasone. Egr-1 protein expression was increased in the cells surrounding the smaller airways in the animals exposed to ventilation (Figure 3). Similar staining patterns were seen in dexamethasone and cortisol groups. Betamethasone exposed animals had fewer Egr-1 positive cells (Figure 3F).

**Figure 4:**
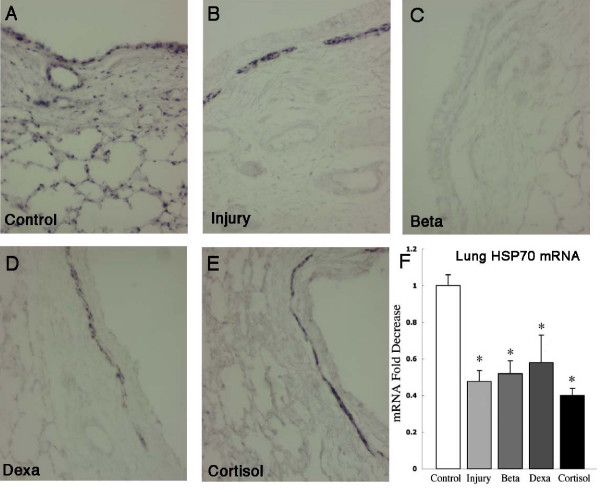
**HSP70 mRNA localization**. (A) HSP70 mRNA was localized to the bronchial epithelium and parenchyma in unventilated controls. (B-E) Ventilation decreased HSP70 mRNA in airway epithelial cells and parenchyma. Injurious ventilation induced HSP70 mRNA in the smooth muscle surrounding large airways in all groups except the Beta group (C). (D) RNase protection assay for HSP70 mRNA in lung demonstrates decrease in lung mRNA with ventilation in all groups. *p < 0.05 vs controls.

Heat Shock protein 70 (HSP70) mRNA is normally expressed by the airway epithelium and some parenchymal cells in fetal sheep [3] (Figure 4). The mRNA decreased in all ventilated groups. The HSP70 mRNA signal was lost from bronchial epithelium with ventilation and induced in the smooth muscle surrounding the larger airways. The Betamethasone group also lost the bronchial epithelium mRNA but there was no induction in the smooth muscle.

### Systemic response to ventilation

All ventilation groups had increased mRNA for the acute phase reactant Serum Amyloid A3 (SAA3) in the liver (Table 2). Antenatal or postnatal steroids did not affect liver acute phase response to initiation of ventilation with large tidal volumes.

## Discussion

Ventilation of preterm lambs, in the alveolar stage of lung development, with escalating V_T _to about 15 mL/kg by 15 min causes activation of a pro-inflammatory cascade in the lung, with both airway and tissue involvement [16,31]. Antenatal treatment with betamethasone decreased the injury score, protein leak, and pro-inflammatory cytokines compared to animals receiving no treatment (Injury), and tended to decrease neutrophils in the BAL. Betamethasone treatment improved lung function after large tidal volume injury and surfactant treatment. Postnatal dexamethasone had variable effects on the pro-inflammatory cytokine production, with a decreased IL-1β and MCP-1 production, but did not prevent the deterioration in lung function by 3 h. The postnatal cortisol treatment had minimal effects under these experimental conditions. The induction of the acute phase reactant Serum Amyloid A3 in the liver was not affected by any of the steroid treatments.

Glucocorticoids mediate their anti-inflammatory effects by activation of the intercellular glucocorticoid receptor (GR). Once activated and released from heat shock protein 90, the GR can translocate into the nucleus and decrease activity of NF-κB and activating protein-1 [18,21]. The receptor can also dimerize and block binding sites for pro-inflammatory transcription factors [18]. A third action of GR is up-regulation the NF-κB inhibitor IκB-α [18]. Finally, the GR can increase levels of cell ribonucleases and mRNA-destablizing proteins [18]. Betamethasone and dexamethasone are potent synthetic fluorinated glucocorticoids. Cortisol has weaker glucocorticoid activity but also has mineralocorticoid activity [32]. The cortisol dose of 2 mg/kg is used by clinicians and has roughly one eighth the anti-inflammatory potency of the dexamethasone dose (0.5 mg/kg)[21]. The equivalent corticosteroid activity (12.5 mg/kg) could have different effects. In our model, both betamethasone and dexamethasone decreased the induction of the pro-inflammatory cytokines MCP-1 and IL-1β. In preterm sheep, the initiation of ventilation leads to IL-1β production from the inflammatory cells and airway epithelium, whereas MCP-1 mRNA was localized to the mesenchyme surrounding the small airways[31]. Although the dexamethasone treatment did not decrease protein in BAL or the inflammatory cells, the decrease in multiple pro-inflammatory cytokines suggests it targeted multiple cell types in the lung.

In adult animal models, ventilation with large tidal volumes leads to pulmonary and systemic responses [33] and these responses can be attenuated by pretreatment with corticosteroids [19,34]. Rats exposed to large tidal volume ventilation had a deterioration in respiratory function, and pretreatment with dexamethasone 30 minutes prior to ventilation decreased both physiologic deterioration and pro-inflammatory cytokines [34]. When ventilated with large tidal volumes, isolated and perfused rat lungs produce pro-inflammatory cytokines and chemokines through a NF-κB pathway that is independent of LPS-TLR4 signaling, and the inflammatory activation is blocked by dexamethasone [19]. Our lambs demonstrated similar increases in cytokines, with partial blockade by dexamethasone. Another glucocorticoid, methylprednisolone, decreased neutrophil activation in rats exposed to large tidal volume ventilation[35]. None of the corticosteroid treatments had a dramatic effect on cellular influx in our studies, though neutrophil function was not tested. The studies in adult animals gave the corticosteroids at an interval before the ventilation injury, and that strategy worked for Betamethasone in these preterm lambs. A treatment with Dexamethasone shortly before preterm birth might also be effective. Cortisol does not cross from the mother to the fetus in sufficient amounts to have any anticipated benefit [36].

The effects of antenatal betamethasone on lung injury from ventilation may be due to activation of the glucocorticoid receptor to suppress inflammation or may result from structural or biochemical changes in the fetal lung that protect the lung from acute lung injury. Lambs exposed to antenatal corticosteroids have thinner alveolar walls, elongation of secondary septa, and an increased alveolar volume [37]. Changes in lung compliance from antenatal corticosteroids are due primarily to alterations in the tissue compartment of the lung rather than the airways [38]. Although lambs given antenatal corticosteroids between 8 and 15 hours prior to delivery have increased lung compliance and decreased edema, lambs do not increase surfactant pools until 4 or more days after maternal treatment [22,39]. Thinning of distal airways were noted qualitatively on histology examination of the betamethasone group. Clearance of airway fluid through activation of sodium transporter by betamethasone also may contribute to the decreased the airway injury seen with initiation of ventilation [40]. Induction of HSP70 mRNA in smooth muscles is likely due to over-distention of airways during initiation of ventilation. Clearance of lung fluid prior to ventilation in the betamethasone group may have resulted in a more even distribution of the tidal volume and less stress on the airways and their smooth muscle. Although antenatal betamethasone can increase antioxidant activity in premature infants[10], we did not evaluate antioxidant effects. The average PaO2 of 30 to 50 mmHG in the lambs throughout ventilation period was sufficient to maintain a saturation >85%. We have previously explored the antioxidant effects in fetal sheep exposed to LPS and only small amounts of oxidants were released [41]. Near-term lambs exposed to 100% oxygen for 3 h also had minimal oxidative damage [42,43]. A recent study showed that betamethasone was as effective as dexamethasone for weaning premature infants from ventilators with fewer short term side effects [44]. Since only a few minutes elapsed between dexamethasone administration and injurious ventilation, there was insufficient time for changes in vascular or alveolar structures. The difference in response to betamethasone and dexamethasone probably resulted from the timing of treatment relative to delivery. If given antenatally, dexamethasone may have had similar effects to betamethasone.

Some preterm infants have a decreased ability to produce cortisol in response to stress and low cortisol levels have been linked to an increased risk of BPD [45]. In infants exposed to chorioamnionitis, early treatment with low-dose hydrocortisone decreased the rate of BPD without an increase in cerebral palsy [14,46]. A small study of prolonged hydrocortisone treatments demonstrated cortisol was as effective as dexamethasone for decreasing FiO_2 _and weaning infants from the ventilator [47]. We did not show an effect of cortisol on acute lung injury from the initiation of ventilation. One of the limitations of the study is the short period of ventilation, and beneficial cortisol effects could appear later.

The use of corticosteroids to treat acute lung inflammation has been studied in multiple human diseases, including cardiopulmonary bypass, ARDS, and bronchiolitis. Corticosteroids given 30 min before bypass decreased levels of the pro-inflammatory cytokine TNF-α, IL-6, and IL-8, and increased expression of the anti-inflammatory cytokine IL-10 [48]. The decrease in pro-inflammatory cytokines was partially attributed to stabilization of IKβ-α, thus preventing NF-κB from nuclear translocation [20]. Dexamethasone prior to cardiac bypass decreased C-reactive protein, but did not effect clinical course or alter the endothelial markers von Willebrand factor antigen or S100b protein [49]. Dexamethasone has a similar effect on pro-inflammatory cytokines, Il-1β and MCP-1 in lambs, without changes in the systemic acute phase reactant SAA3 in the liver. Similar to studies of corticosteroids prior to cardiac surgery, we found no difference in physiology or degree of inflammation in the lungs. In adults, corticosteroids may improve survival with ARDS, but there is an increased risk of ARDS or mortality when corticosteroids are given in a preventative manner [50]. The increased pro-inflammatory risk of preventative corticosteroids in ARDS may be due to upregulation of cytokine receptors in response to corticosteroids [51]. In the setting of moderate to severe RSV bronchiolitis, dexamethasone treatment did not improve outcomes [52,53]. The routine use of dexamethasone in the setting of acute lung injury requires further study.

## Conclusions

Initiation of ventilation with large tidal volumes leads to lung injury and systemic inflammatory responses. Although antenatal betamethasone treatment decreased the lung injury and improved ventilation, lung inflammation and systemic changes in acute phase responses in the liver still occurred. Our results support the use of antenatal corticosteroids treatments to decrease mortality and morbidity in preterm infants. A better tolerance to the initiation of ventilation may contribute to the pleiotropic benefits of this therapy. The use of anti-inflammatory medications for chronic inflammation merits further exploration, though short term use in the acute setting may be of no benefit. Procedures to decrease the use of mechanical ventilation and to minimize volutrauma in the delivery room should include antenatal corticosteroids, but treatment with corticosteroids at birth is not supported by our results.

## Competing interests

The authors receive grant and equipment support from Fisher & Paykel Healthcare, Auckland, NZ. to perform neonatal resuscitation research. Chiesi Faraceuticals, S.p.A provided a gift of surfactant. All experiments were designed and analyzed by authors.

## Authors' contributions

NH did animal care, tissue processing, analysis, and manuscript preparation. JJP did animal care, experimental design, analysis, and manuscript preparation. MB did animal care. GP did animal care, maternal sheep injections. SK did experimental design, tissue analysis, and manuscript preparation. AJ did experimental design, tissue analysis, manuscript preparation, and received NIH funding.
